# A multi therapy bioelectronic wound dressing

**DOI:** 10.1038/s44385-026-00081-x

**Published:** 2026-04-07

**Authors:** Kaelan Schorger, Hsin-ya Yang, Sujung Kim, George Luka, Shaamil Pirzada, Prabhat Baniya, Anthony Gallegos, Willie Feng-Liu, Maryam Tebyani, Sydnie Figuerres, Kevin Jiang, Houpu Li, Celeste Franco, Elham Aslankoohi, Jaime Tenedorio, Athena M. Soulika, Mircea Teodorescu, Roslyn Rivkah Isseroff, Marco Rolandi

**Affiliations:** 1 Department of Electrical and Computer Engineering, University of California, Santa Cruz, Santa Cruz, CA USA; 2 Department of Dermatology, School of Medicine, University of California, Davis, Davis, CA USA; 3 MedTec Consulting, Woodside, CA USA; 4 Pediatric Regenerative Medicine, Shriners Hospitals for Children, Sacramento, CA USA

**Keywords:** Biological techniques, Biotechnology, Health care, Medical research

## Abstract

Chronic and acute skin wounds affect more than six million people in the United States each year. Many heal with basic care, but others do not and become infected, scarred, or chronic. These wounds reduce quality of life and increase healthcare costs. Bioelectronic integrated wound dressings and smart bandages can improve healing by delivering treatments locally and on demand, including electric field therapy and the release of pharmacological compounds. Localized bioelectronic delivery improves healing outcomes and reduces off-target effects. Here, we demonstrate a flexible bioelectronic wound dressing that combines electric field therapy and drug delivery, employing integrated microfluidics to switch between therapeutic modalities. Treatment with the bioelectronic dressing in a pilot porcine wound study showed promising results, including increased wound closure rates, improved tissue maturity, and reduced inflammatory response, compared with standard of care.

## Introduction

Chronic and acute skin wounds affect more than 6 million patients in the US annually^1^. Many wounds heal with basic care; others require timely medical treatment^2^. Failed or delayed treatment can lead to infection, scarring, and chronic non-healing wounds^3^. Wounds that do not heal are a significant source of morbidity^4^, decrease quality of life^5^, and present an increasing burden on the healthcare system^6^.

To improve treatment outcomes, portable bioelectronic devices, wearables, and smart bandages enable wound treatment^7^ with microneedles^8,9^, phototherapy^10^, and wireless bandages with sensing and drug delivery functions^11,12^. These bandages enhance or redirect the natural healing processes that internal factors, such as diabetes, or external factors, such as bacterial infection, may impair^13^. Wound dressings with integrated bioelectronics can combine electric-field (EF) stimulation^14,15^ with active, localized, on-demand delivery of therapeutic agents^11,12^.

EFs play a critical role in the wound healing process. Upon skin injury, disruption of the transepithelial potential generates a lateral endogenous EF, typically 40–200 mV/mm^16^. This field directs keratinocytes through galvanotaxis, enhances cell proliferation at the wound edge, and promotes coordinated epithelial cell migration during re-epithelialization^17–25^. Over the past two decades, EF-generating wound treatment devices have been shown to increase re-epithelialization, accelerate wound closure, and reduce infection^14,15,26,27^.

Localized, bioelectronic delivery of therapeutic agents increases bioavailability and reduces off-target effects compared to systemic or topical delivery^28^. An externally applied EF drives charged molecules directly into the tissue^29^, precisely controlling the dose, rate, location, and timing. Delivery via charge transfer is advantageous because it can maintain sustained delivery at specific rates over time and interface with electronics for adaptive control.

Bioelectronic delivery expands the therapeutic possibilities of a range of compounds that may have limited application in wound healing when delivered conventionally, but can have significant clinical utility when delivered with more exact and responsive methods. One such drug is Fluoxetine (FLX), a common serotonin reuptake inhibitor that has shown significant utility in modulating inflammation and improving wound closure rates^30–33^. Our group has previously enhanced wound healing in murine and porcine models with programmable and machine learning driven adaptive bioelectronic EF and FLX treatment^34–37^. From a bioelectronics and materials perspective, fluoxetine is particularly well-suited for integration with EF. FLX is a charged small molecule whose transport can be precisely controlled using iontophoretic bioelectronic delivery, enabling spatiotemporal dosing that is difficult to achieve with topical or systemic administration. Prior work has shown that endogenous wound EFs can regulate keratinocyte migration, immune cell trafficking, and re-epithelialization, and applied EF therapy has been shown to accelerate wound closure and reduce inflammation^14,15,26,27^. Programmable EF delivery and bioelectronic fluoxetine delivery modulate complementary aspects of wound biology, providing a rational basis for their combined evaluation within a single flexible platform.

**Fig. 1:**
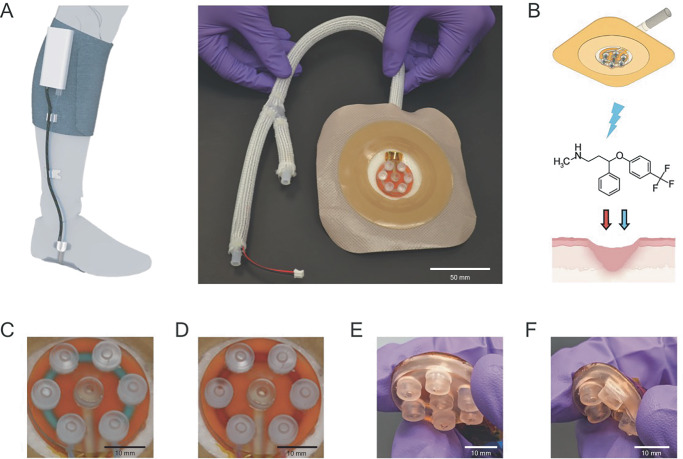
Flexible bioelectronic wound dressing. **A** Conceptual illustration of clinical application (left) and the wound contacting portion of flexible multitherapy bioelectronic dressing (right). **B** Illustration depicting EF therapy and FLX Delivery. **C** Actuator of bioelectronic bandage filled with blue dye representing EF solution. **D** Actuator of bioelectronic bandage filled with red dye representing FLX. **E** Bioelectronic bandage actuator before bending. **F** Bending of the bioelectronic dressing actuator.

Here, we expand on this previous work with a flexible active wound dressing capable of conforming to diverse anatomical topologies and delivering multiple therapies in sequence through bioelectronic delivery and integrated microfluidics [Fig. 1]. We characterize the device deploying both EF therapy and FLX delivery and demonstrate its wound-treating efficacy in a porcine model, which closely mimics human skin architecture and wound healing kinetics^38^. Results from our in vivo testing demonstrate that the bioelectronic dressing accelerates wound closure while reducing granulation tissue and inflammation response.

## Results

### Bioelectronic bandage design

The design of the bioelectronic dressing provides flexibility and delivers two distinct time-dependent therapies to the wound bed [Fig. 1]. The device has two parts. The outer case holds the controller, batteries, and pump module, while the dressing consists of a flexible bioelectronic actuator embedded in a traditional wound dressing. This design makes the dressing easier to place on curved anatomy with complex topology, like the bottom of the foot, where chronic wounds often appear [Fig. 1A]. Separating the dressing portion of the bioelectronic bandage from the control board and pump module keeps the wound-contacting portions of the dressing soft and flexible while enabling digital control and on-demand changes in therapeutic type. The dressing uses bioelectronic actuation to deliver EF or FLX therapy to the wound site. The dressing employs an internal microfluidic channel, molded into the main PDMS body of the device, connected to microfluidic tubing [Fig. 1B] that allows different solutions to be pumped into and out of the device, enabling a single device to deliver multiple therapy types [Fig. 1C, D].

To maximize contact between the wound and the ion-conducting elements of the device, the bioelectronic bandage has eight protrusions [Fig. 1E]. These protrusions contain an AMPSA-PEGDA hydrogel that serves as the interface between the solution containing the therapy and the wound [Fig. 1E]. The specific design allows the bandage to be flexible, maintaining the integrity of the ion-selective hydrogel [Fig. 1F].

While the AMPSA-PEGDA hydrogel previously characterized by our group exhibits good ion transport and selectivity^39^, it is highly brittle and difficult to adhere to surfaces. To mitigate this, we developed a method for progressively curing the hydrogel in several layers within a double-fluted plug shape, molded within PDMS protrusions (Text S1). The double-fluted shape allows the hydrogel to be cured in the open-ended protrusion (which must contact both the fluidic channel and the wound bed) without sliding out, relying on mechanical stability rather than surface chemistry.

**Fig. 2:**
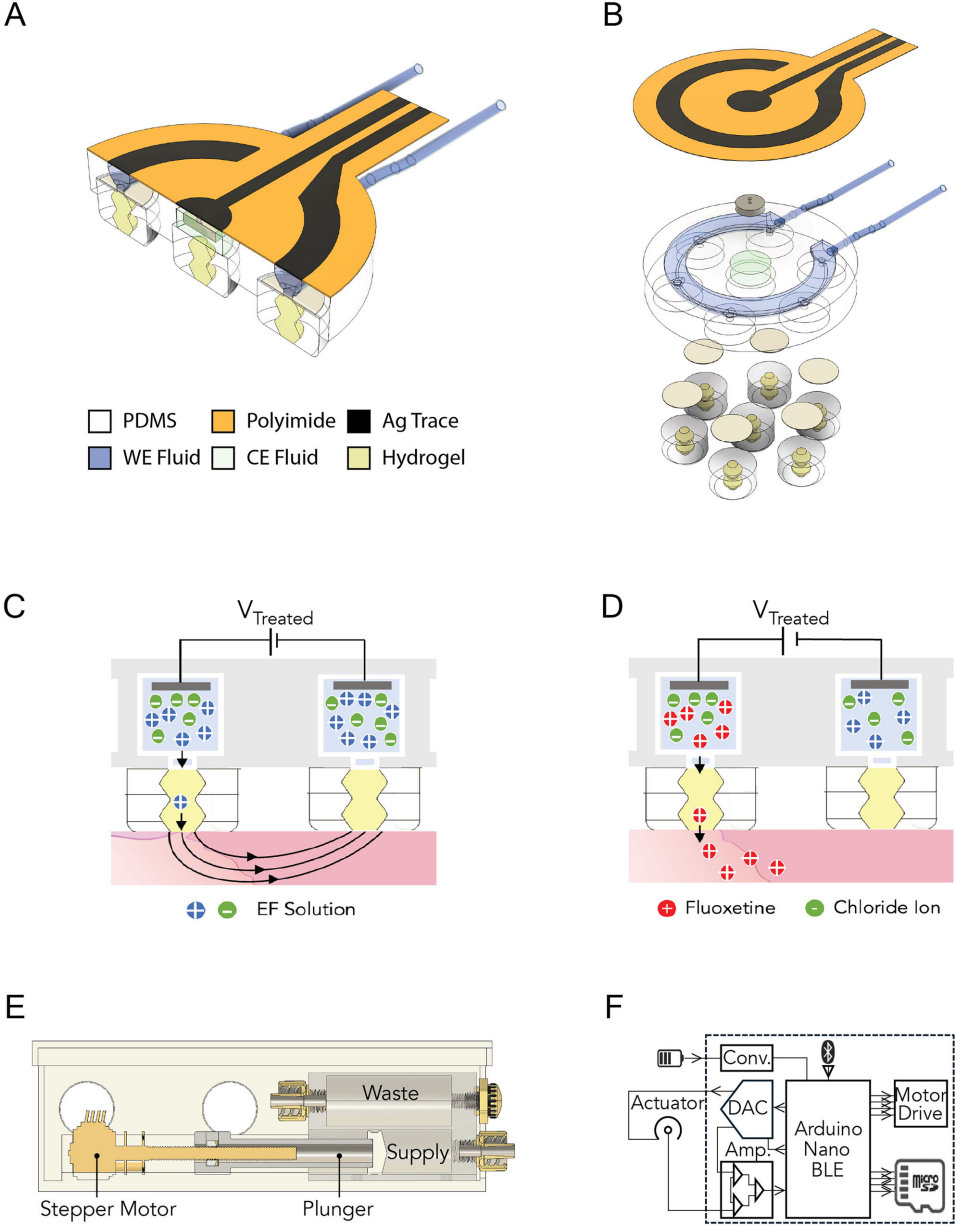
Flexible bioelectronic wound dressing design and components. **A** cross-sectional view of the bandage actuator. **B** Exploded view of bandage actuator showing cellulose acetate membranes above PDMS protrusions and AgCl pellet electrode above actuator main body. **C** Bioelectronic EF generation. **D** Bioelectronic FLX delivery. **E** Diagram of pump module. **F** Control board diagram.

The actuator portion of the dressing consists of a flexible polyimide electrode with silver traces, a PDMS body with an internally molded fluidic channel, cellulose acetate membranes, PDMS protrusions, microfluidic tubing connecting the actuator to the rest of the fluidic system, and a ribbon connector that joins wiring from the control board to the polyimide electrode [Fig. 2A, B]. Each PDMS protrusion contains a cation-selective AMPSA-PEGDA hydrogel that acts as the ion exchange membrane [Fig. 2A]. This ion-exchange membrane relies on fixed negative charges in the hydrogel structure, allowing only positively charged ions to transfer from the reservoir to the wound bed^39^. A cellulose acetate membrane separates the hydrogel from the microfluidic channel, minimizing potential contamination of the reservoir with wound fluid. The polyimide electrode has two silver traces inkjet printed with silver nanoparticle ink. The outer ring functions as the working electrode (WE) trace, and the inner acts as the counter electrode (CE) [Fig. 2A]. The purpose of this configuration is to create a cathodic wound center during EF therapy and to allow drug deposition at the wound edge during FLX treatment. Behind the center plug, there is a fixed fluidic reservoir and an Ag/AgCl pellet, which is connected to the CE trace of the polyimide electrode [Fig. 2B]. The actuator employs an internal microfluidic channel, molded into the main PDMS body of the device, connected to microfluidic tubing that allows different solutions to be pumped into and out of the device, enabling a single device to deliver multiple therapy types.

The basic mechanism of the bioelectronic actuator [Fig. 2C, D] is the iontophoretic delivery of positive cations from the microfluidic reservoir containing to the wound bed^34–37^. We define the voltage between the WE and the CE as *V*_treat_ for EF and FLX treatment, respectively. For positive *V*_treat_, EF treatment occurs when the microfluidic reservoir contains a saline solution that facilitates augmenting the endogenous EF already present in the wound bed^39^ [Fig. 2C]. Induced EF reinforces the endogenous EF of the wound bed and serves to direct migration of keratinocytes, macrophages, and neutrophils through galvanotaxis^15^. The direction of these cell types to the wound center expedites the transition from the inflammatory to the proliferative healing phase^40^.

To switch to fluoxetine therapy, 10 mM fluoxetine hydrochloride solution is transferred into the device’s fluidic lines and channel by the pump module [Fig. 2E]. Inspired by open-source insulin pumps^41^, the pump module consists of an outer enclosure box that contains a high-reduction stepper motor, a plunger arm, and a dual-chamber reservoir. Turning the stepper motor drives the plunger arm forward inside the internal sidewall rails of the enclosure. The plunger arm pushes a piston inside the supply reservoir, moving 10 mM fluoxetine hydrochloride solution out the luer fitting at the opposite end of the supply chamber, connecting to the microfluidic inlet tubing of the device. The fluid being pumped through the device’s outlet tubing returns to the pump module and into the waste reservoir. A breather vent is attached to the opposite end of the waste reservoir, allowing air to escape as solution is pumped in.

The device’s control board consists of an Arduino Nano Bluetooth Low Energy (BLE) microcontroller, a buck converter, a DAC, an instrumentation amplifier, a micro-SD breakout board, and a stepper motor controller [Fig. 2F]. This control enables us to set specific currents via PID voltage control, monitor device output in real time via BLE, and store delivery data locally. The stepper motor controller drives the stepper motor in the pump module, allowing the microcontroller to dictate flow through the microfluidic system.

### Delivery strategy and in vitro characterization

**Fig. 3:**
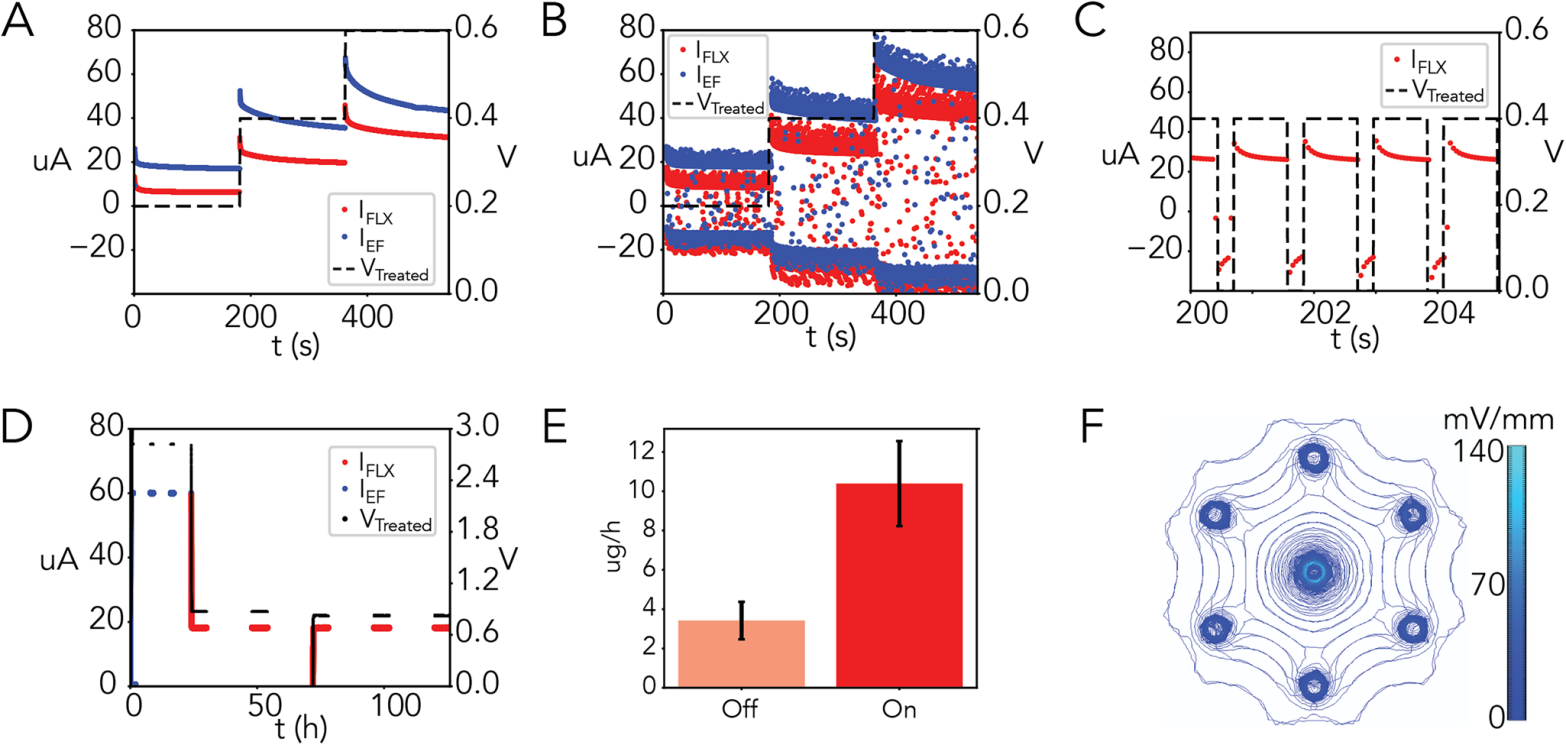
In vitro characterization of flexible bioelectronic wound dressing. **A** Constant voltage ${V}_{\mathrm{treat}}$ current response for device delivery of fluoxetine and EF of the device in the Steinberg solution. **B** 77% duty cycle pulsed ${V}_{\mathrm{treat}}$ current response of the device in the Steinberg solution. ${V}_{\mathrm{treat}}$ is depicted as the peak of the pulse. **C** Zoomed in view of the current and voltage pulse for fluoxetine delivery. **D** Control board resistor test (47k Ω) for 6-day treatment cycle. **E** HPLC results for on/off ratio testing of fluoxetine delivery (*n* = 5). Mean delivery rate for devices while “on” (target = 20 μA) was 10.40 μg/h, while “off” devices diffused fluoxetine at an average rate of 3.42 μg/h. **F** COMSOL EF simulation of device on wound bed. Magnitudes of 40 mV/mm are found at the outer WE hydrogel contact points, while magnitudes of 100 mV/mm are reached at the central CE hydrogel contact point.

Before testing the bioelectronic bandage in vivo, we characterized the system in vitro in Steinberg solution^42–45^ to provide a consistent ionic environment for benchtop testing (Fig. 3). FLX+ ions have a lower ion mobility than the ions we use for EF generation (Na+, K+) and thus have a lower current when delivered with an equivalent constant voltage ${V}_{\mathrm{treat}}$ [Fig. 3A]. Pulsing ${V}_{\mathrm{treat}}$ results in larger peak currents at equivalent peak voltage [Fig. 3B, C] [Text S1].

To provide periodic EF therapy during the immediate inflammation stage following wounding, the device is programmed to deliver EF for 1 h every 8 h during the first 24 h following surgery. To achieve sustained fluoxetine concentration at the wound site beyond the first 24 h of inflammation, fluoxetine was iontophoretically delivered at a target rate of 10 μg/h for 6 h each day for the subsequent 5 days following the 24 h EF treatment. EF therapy is employed first to maximize its benefit during the inflammation stage^46^, while fluoxetine is delivered during late inflammation and subsequent stages of wound healing^32^. While fluoxetine delivery can help hasten the transition from inflammation to proliferation healing stage, when employed too early, it may impair wound healing by curtailing the beneficial and necessary aspects of the inflammation process^47^. A 6-day delivery program resistor test of the control board is presented in Fig. 3D, with EF therapy during the first day shown in blue and subsequent fluoxetine delivery shown in red.

HPLC characterization of active bioelectronic fluoxetine delivery demonstrates an effective on/off ratio of 3:1 [Fig. 3E]. During the “off” cycle of the device, *V*_treat_ is set at −0.1 V to minimize FLX diffusion into the wound bed. COMSOL simulation of EF generation [Text S1] showed an estimated field strength of 40 mv/mm at the outer protrusion contact points and 100 mv/mm at the center CE protrusion contact [Fig. 3F].

### In vivo testing in a porcine wound model

**Fig. 4:**
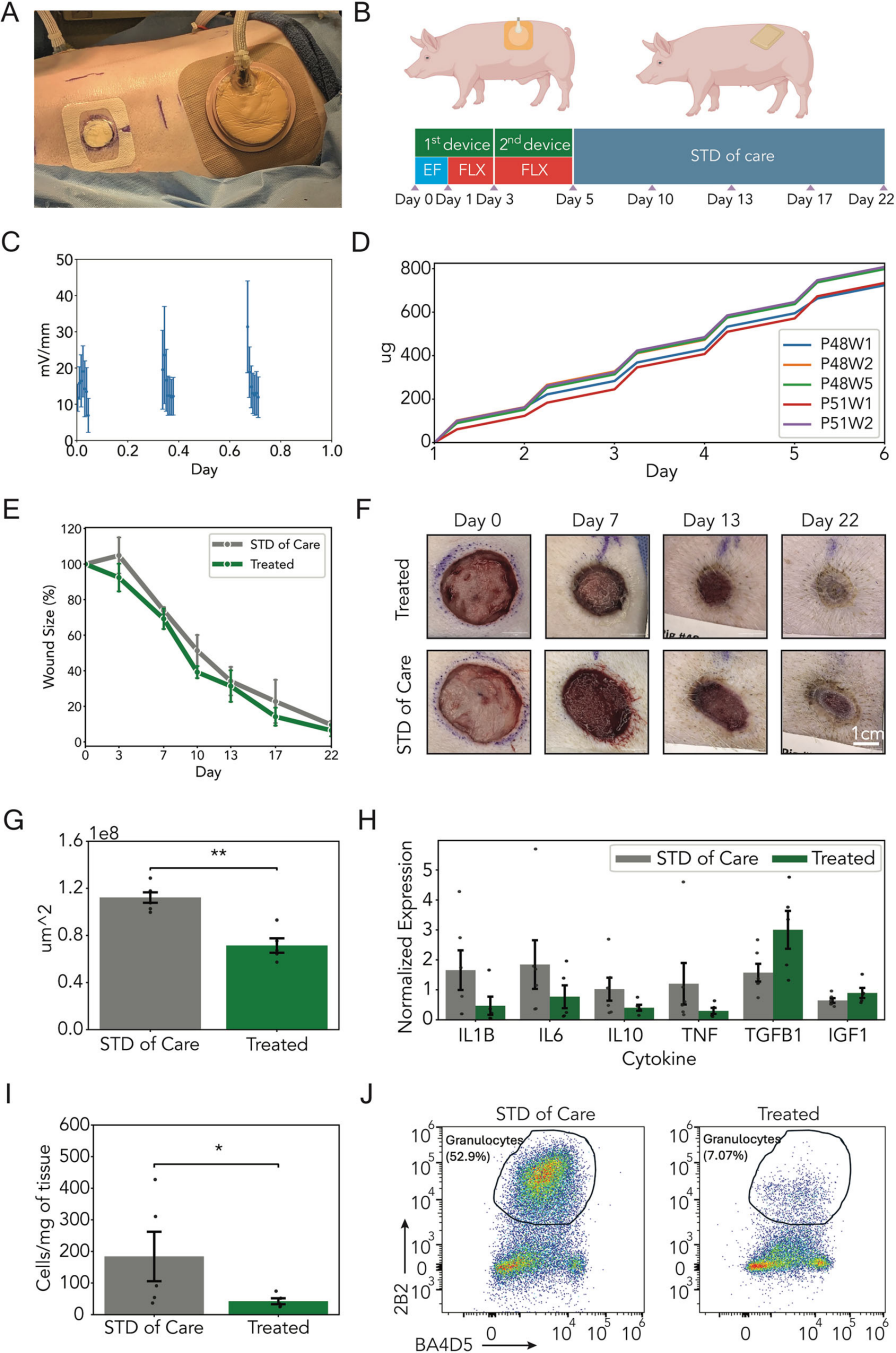
In vivo deployment of flexible bioelectronic wound dressing. **A** Control wounds dressed with STD of care (left) and wounds treated with bioelectronic wound dressing (Treated), (right) after day 0 wounding procedure. **B** Treatment plan for wounds treated with the bioelectronic bandage. **C** Average device applied EF strength of device therapy from WE hydrogel wound contact to CE hydrogel wound contact with SEM bars. **D** Estimated cumulative delivered fluoxetine dosage in device-treated wounds accounting for both active delivery and passive diffusion. Each color again represents an individual wound. **E** Average wound area expressed as a percentage of original size across over the course of the 22-day experiment with SEM. Wounds treated with the bioelectronic bandage (Treated) demonstrated consistently reduced wound size compared to control, but this difference failed to reach statistical significance on any single day of measurement. However, statistical analysis across all experimental days showed a statistically significant difference between treated and standard of care groups with regard to wound size (*p* = 0.0145, two-way ANOVA; ${n}_{\mathrm{SOC}}=6$, ${n}_{\mathrm{device}}=5$). **F** Representative wound images for Treated and control wounds. **G** Tissue granulation area from histological analysis. Device-treated wounds showed a 36.3% reduction compared to SOC control (*p* < 0.005). **H** Normalized cytokine expression obtained from qPCR showing decreased inflammation-associated cytokines (IL1B, IL6, IL10, TNF) and increased pro-reparative cytokines (TGFB1, IGF1) in device-treated wounds compared to control. Device-treated wounds had a 71.7% reduction in average expression of IL1B (*p* = 0.18), a 58.2% reduction in IL6 (*p* = 0.25), a 60.8% reduction in IL10 (*p* = 0.33), and a 75.4% reduction in TNF (*p* = 0.18). Device-treated wounds showed an increased average 91.1% increase of TGFB1 (*p* = 0.08) and a 39.2% increase in IGF1 (*p* = 0.18). **I** Flow cytometry quantification showing a 60.0% decrease of granulocytes in device-treated wounds (*p* = 0.0278). **J** Representative flow cytometry quantification of granulocytes.

To assess the effectiveness of the bioelectronic bandage in a validated model, we conducted in vivo testing on two porcine specimens^38^. Each of the two porcine models received six excisional 30 mm diameter, 7 mm deep, partial thickness wounds along the back. In each porcine model, three wounds were treated with the bioelectronic dressing (referred to here as “treated”) and three wounds were dressed with standard of care as a control [Fig. 4A]. The healing progression of the wounds was monitored over the course of 22 days. Standard of care (STD) consisted of a Tegaderm 30 mm wound veil and 30 mm Optifoam bolsters. Across both swine, this arrangement yielded six device-treated wounds and six control wounds. Device-treated wounds (treated) were treated with the bioelectronic dressing from postoperative day 0 to postoperative day 5. Bioelectronic dressings were replaced with standard-of-care bandages after 3 days (postoperative day 3). After postoperative day 5, device-treated wounds were dressed identically to standard-of-care wounds. Subsequent bandage changes occurred on postoperative days 7, 10, 13, and 17 [Fig. 4B]. During each bandage change, wounds were cleaned and photographed. At day 22, the porcine specimens were euthanized, and tissue was collected for histology.

One device was entirely removed by one of the porcine models during unobserved free roaming before the start of treatment on the second postoperative day and was excluded from the study. However, the remaining device-treated wounds successfully received therapy from functioning actuators, pump systems, and control boards, resulting in a bioelectronic dressing-treated group of *n* = 5 and a control standard-of-care group of *n* = 6. Wounds showed no visible signs of infection at the end of the study (day 22).

Average EF strength, calculated from in vivo current data and model-derived wound-be resistance, shows the average EF reaching a maximum of 80 mV/mm during in vivo operation [Fig. 4C]. The total cumulative dose over the 5 days of FLX treatment, accounting for both active delivery and passive diffusion, was estimated based on in vitro delivery rates measured with HPLC and delivery currents measured by the devices during the in vivo experiment. These values across all devices range from 723 to 808 μg, with a mean cumulative dose of 773 μg ([Fig. 4D]). This results in a mean daily dose of 154.6 μg/day.

Wounds treated with the bioelectronic dressing were consistently smaller (expressed in terms of original area) than their standard of care counterparts (*p* = 0.0145, two-way ANOVA), when analyzed across all postoperative days of the experiment [Fig. 4E]. Representative wound images are depicted in [Fig. 4F]. A full matrix of wound images depicting all wounds across all imaging days is shown in Fig. S1.

As healing progresses, granulation tissue transforms into mature oriented collagen fibers, dense vasculature recedes, and dermis is regenerated. Although the complete reorganization of the dermal/collagen network takes months, at the endpoint (day 22) of these porcine experiments, we observed partial resolution of the granulation tissue [Fig. S2]. Granulation area was 36.3% smaller in wounds treated with the bioelectronic dressing (*p* < 0.005) [Fig. 4G], indicating device-treated wounds matured faster^48^.

Analysis of individual cytokines revealed a consistent pattern in wounds treated with the bioelectronic dressing, with reductions in inflammation-associated cytokines (IL1B, IL6, IL10, TNF) and increases in pro-reparative cytokines (TGFB1, IGF1) compared with standard of care. Although none reached statistical significance after FDR correction, TGFB1 trended strongly toward higher expression in the device group (permutation *p* = 0.060). To assess the overall cytokine profile, we applied combined probability tests across the six inflammatory and reparative markers. This analysis demonstrated global significance (Fisher’s test *p* = 0.048; Stouffer’s test *p* = 0.012), supporting the conclusion that the device alters the cytokine milieu toward reduced inflammation and enhanced repair, despite the limited power of individual comparisons [Fig. 4H].

When assessed by flow cytometry^49^, wounds treated with the bioelectronic bandage had significantly reduced numbers of granulocytes compared to the standard of care [Fig. 4I]. In this context, these granulocytes are mostly presumed to be neutrophils, a pro-inflammatory white blood cell type [Fig. 4J]. Standard-of-care control wounds had an average granulocyte count of 35.7 cells/mg of tissue, while bioelectronic dressing-treated wounds had an average count of 14.3 (2B2+, *p* = 0.0278). This reduction further demonstrates the bioelectronic dressing’s inflammation-modulating effects.

## Discussion

Wounds treated with the bioelectronic dressing showed improvements across a range of wound-healing metrics compared with standard-of-care controls. Image-based wound size analysis showed an apparent reduction in wound size in device-treated wounds [Figs. 4E and S1]. Higher average re-epithelialization and Rete peg counts in bioelectronic dressing-treated wounds further support this conclusion [Fig. S3]. Additionally, wounds treated with the bioelectronic dressing also showed advanced healing maturity, as evidenced by reduced granulation tissue at day 22 [Fig. 4G] and reduced inflammation, as demonstrated by cytokine expression [Fig. 4H], and lower quantities of granulocytes [Fig. 4I].

While porcine models provide one of the closest analogs to human healing conditions, adapting or developing devices intended to be worn for multiple days at a time by pigs can involve sacrificing a certain amount of device subtlety and delicacy in exchange for robustness. Future work developing similar devices would investigate more self-contained patch-like integrations of microfluidic systems inside the dressing itself and designs capable of treating a wider range of wound sizes with a single device. Reconciling the design criteria of portable treatment devices worn for long periods of time by animal models with the more delicate attributes and form factors required for true translational clinical application remains a challenge.

In this paper, we have demonstrated the viability of a flexible bioelectronic wound dressing capable of delivering two distinct therapeutic interventions via integrated microfluidics and active bioelectronic delivery. The bandage was characterized in vitro and demonstrated functional utility in vivo. Porcine wounds treated with the flexible bioelectronic dressing showed reductions in wound area, increased tissue maturity, and accelerated healing progression compared to standard-of-care-treated wounds. This work presents promising pilot results for the treatment methodology and engineering approach employed.

## Methods

### Bioelectronic bandage fabrication

Actuator main bodies were made from PDMS (Sylgard 184, 10:1) cured inside cutaway resin printed molds (Formlabs Model3 Resin). Electrodes were inkjet printed (Epson XP-15000) using NovaCentrix, JS-A010ET Silver Nanoparticle Ink, 30%w/w Ag ink on a polyimide substrate and cured at 250 °C for 2 min. Next, an Ag/AgCl pellet (World Precision Instruments EP4) is inserted into the CE trace of the electrodes. PDMS main actuator bodies were then cut out of their molds and bonded to the electrodes using an ISO-10993 compliant epoxy (Loctite M-31CL). The tail of the electrode trace was then attached to an FFC connector (DigiKey 609-2158-ND) for integration with harness wiring. PTFE tubing is then inserted into the main body channel connection (Darwin Microfluidics BL-PTFE-1608-50) and wiring attached to the FFC connector.

The anionic hydrogel used as our ion exchange membrane consists of 2-acrylamido-2-methylpropane sulfonic acid (AMPSA) and poly(ethylene glycol) diacrylate (PEGDA), and a UV photo initiator (2-hydroxy-4′-(2-hydroxyethoxy)-2-methylpropiophenone). Protocols and characterization of this hydrogel have been previously reported^39^. Demolded PDMS protrusions are then demolded and placed in a resin-printed curing fixture (Formlabs BioMed Clear Resin). 20uL of hydrogel is then injected into the protrusions and given 4J of UV energy inside a UV crosslinker. This process is repeated, progressively curing the hydrogel in the double fluted plug shape molded in the PDMS protrusion. More information on bandage fabrication, control board, pump module, and actuator fabrication is outlined in Text S1.

### Porcine model

Female Yorkshire-Landrace-Duroc pigs (30–55 kg) were housed and acclimated for 7 days before surgery. During this acclimation phase, pigs underwent habituation to handling procedures, salivary sampling, and harness fitting to reduce stress and improve compliance during the experiment. On the day of implantation, animals were anesthetized, and physiological parameters were continuously monitored throughout the procedure. Blood samples were collected prior to surgery, and the dorsal skin was thoroughly sterilized. All animal experiments were conducted under the protocol approved by the University of California, Davis Institutional Animal Care and Use Committee.

The animal was fasted overnight (12+ hours, no food but water is allowed) prior to the wounding surgery, bandage changes, and the endpoint. A dose of Telazol (5.5 mg/kg via intramuscular injection) was administered for anesthetic induction, followed by masked inhalation of Isoflurane (1–5% to effect). Vital signs were monitored intraoperatively for heart rate, and respiratory rate every 15 min, and body temperature every 30 min. Each pig received six circular partial-thickness 30 mm diameter wounds on its back [Fig. S5]. Wounds were assigned to either receive standard wound care or be fitted with the bioelectronic dressing. Pump modules, control boards, and batteries were secured and placed in bags attached to the harness worn by the animal [Fig. S8]. Post-surgical care included appropriate analgesia to ensure animal comfort and facilitate healing. Daily health and wound checks were performed, with attention to both wound condition and device function. Dressings and bioelectronic modules were replaced every 3–4 days to maintain hygiene and device performance. The connecting wires from the devices were secured in the white tube sleeves and connected to the power banks in the shoulder pouches. The wounded area was further protected by a purple spandex jacket. The total weight of the wound devices was limited to 10% of the body weight of a pig as approved by the IACUC protocol. The total weight of the experimental apparatus for 4 devices, 4 sets of wires, 2 power banks, and 2 pouches is approximately 2 kg on a 40–45 kg pig, lower than <5% of body weight. The pigs were also behaviorally trained pre-operatively, and were examined by the campus veterinarians daily to ensure that there was no sign of pain or distress resulting from the device application during the post-operative period. The pig’s appetite, movements, and weight gain (7–10 lbs/week) were normal during the experiments. At the study endpoint, prior to euthanasia, the animal was anesthetized as described for blood collection and then euthanized with >100 mg/kg pentobarbital via intravenous injection. After euthanasia, a second blood draw was conducted for biochemical assessments.

### Wound analysis

Wound area was calculated using ImageJ and wound images taken immediately after wounding and at each bandage change. Distance to pixel correlation was made using rulers placed by wounds during image collection. Flow cytometry, histological staining, and qPCR were performed to acquire additional biological data [Text S1].

## Supplementary information

Supplementary information

Supplementary

## Data Availability

The data that support the findings of this study are available from the corresponding author upon reasonable request.
