# Supplementary material for: A multi therapy bioelectronic wound dressing
Source: NPJ Biomed Innov. 2026 Apr 7;3:26. doi: 10.1038/s44385-026-00081-x (PMC13055043; doi:10.1038/s44385-026-00081-x)
Supplement: Supplementary file 1 — Supplementary information [file 44385_2026_81_MOESM1_ESM.pdf]

Supporting Information

A Multi Therapy Bioelectronic Wound Dressing

*Kaelan Schorger, Hsin-ya Yang, Sujung Kim, George Luka, Shaamil Pirzada, Prabhat Baniya, Anthony Gallegos, Willie Feng-Liu, Maryam Tebyani, Sydnie Figuerres, Kevin Jiang, Houpu Li, Celeste Franco, Elham Aslankoohi, Jaime Tenedorio, Athena M. Soulika, Mircea Teodorescu, Roslyn Rivkah Isseroff, Marco Rolandi**

K. Schorger, S. Kim, G. Luka, P. Baniya, W. Feng-Liu, M. Tebyani, S. Figuerres, K. Jiang, H. Li, C. Franco, E. Aslankoohi, M. Teodorescu, M Rolandi
Department of Electrical and Computer Engineering, University of California, Santa Cruz, CA 94030 (USA)
E-mail: mrolandi@ucsc.edu

H. Yang, A. Gallegos, R. R. Isseroff
Department of Dermatology, School of Medicine, University of California Davis, Davis, CA 95616, USA.

J. Tenedorio
Medtec Consulting, Woodside, CA 94028, USA.

A. M. Soulika
Pediatric Regenerative Medicine, Shriners Hospitals for Children, Sacramento, CA 95817, USA.

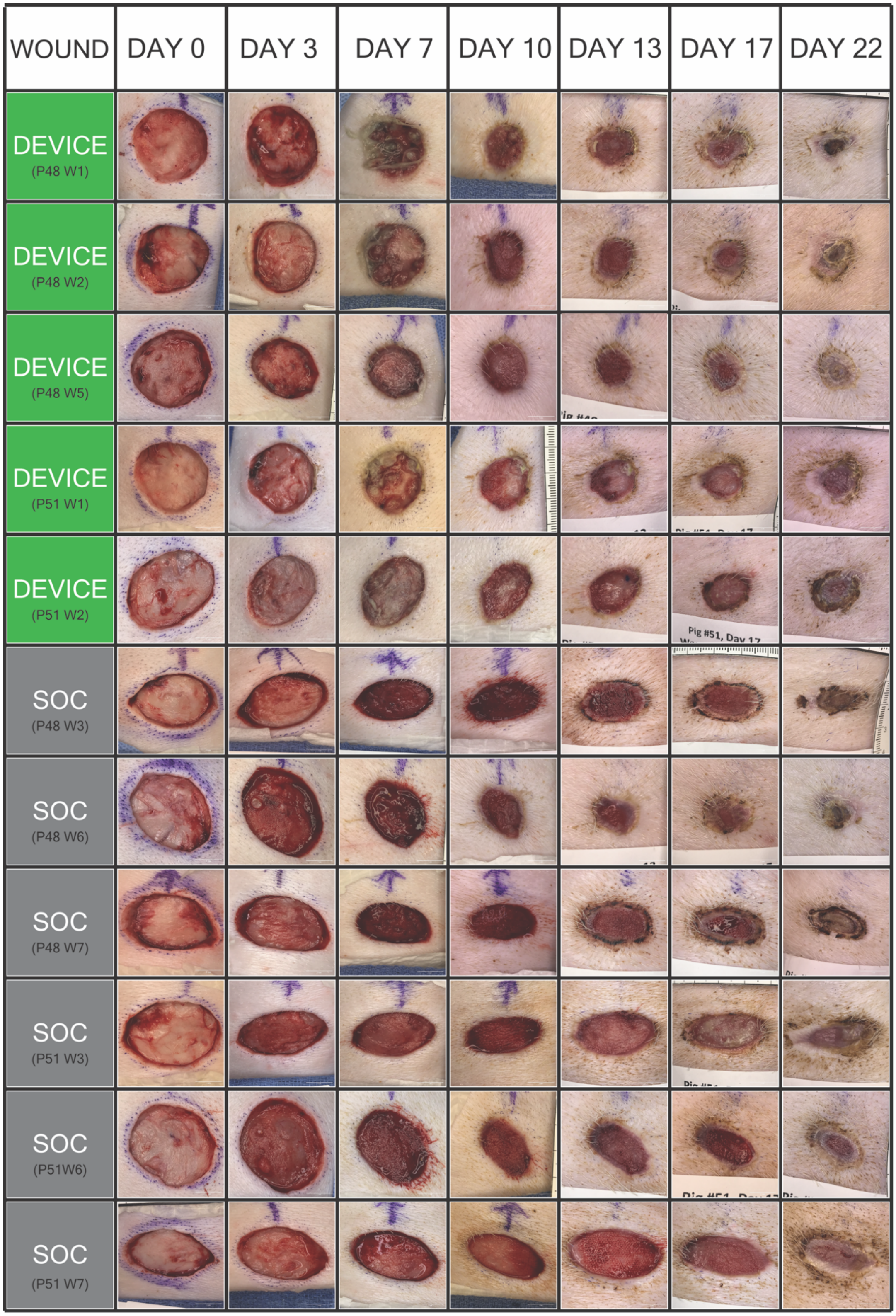

Figure S1. Wound matrix depicting all wounds in experimental data set, across the 22-day duration. All photos are proportionally cropped and scaled to a 4cm-by-4cm area.

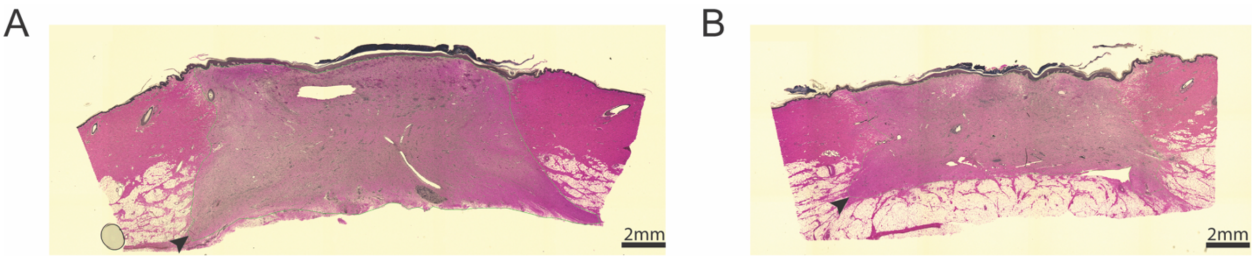

Figure S2. *(*A) Representative histology image of wound treated with standard of care. Granulation area is outlined by a thin green line with an arrow marking the bottom left corner. (B) Representative histology image of wound treated with bioelectronic dressing. Granulation area is again outlined by a thin green line with an arrow marking the bottom left corner.

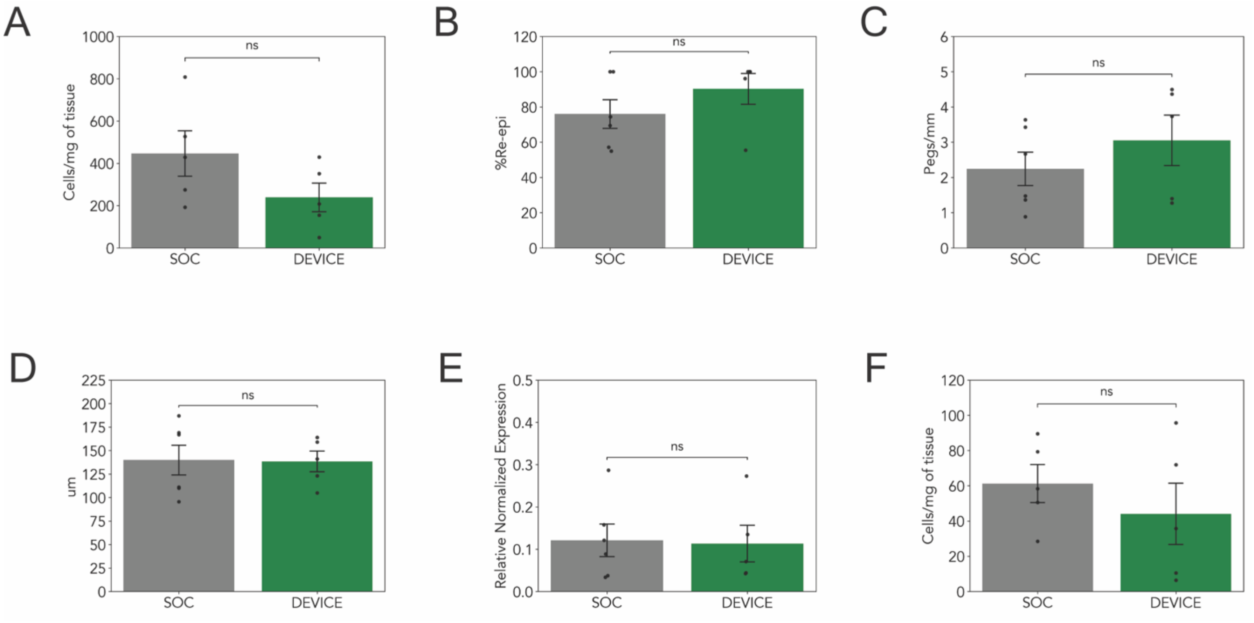

Figure S3. *(*A) From flow cytometry an average decrease of 46.6% in immune cell presence (CD45+) is observed in bioelectronic dressing (DEVICE) treated wounds compared to SOC (p=0.11). (B) Device treated wounds showed a 14.3% increase in re-epithelialization compared to SOC at day 22, but this result lacks statistical significance (p=0.263) (C) Rete pegs are protrusions of epithelium into dermis to enhance the strength and connection of the 2 layers of tissue in healthy, intact skin. Rete pegs gradually regenerated when the epithelium re-surfaces the wound bed and differentiates to multiple layers, another indication of wound resolution. DEVICE treated wounds exhibited a mean increase of 26.6% in Rete pegs/mm (p=0.353) (D) Neo-epithelium expands to multi-layered keratinocytes at the late phase of healing and tissue remodeling, and thickness of the new epithelium can be utilized as another indicator of wound resolution. Neo-epithelium thickness of the neo-epithelium was measured at 12 locations across the wound bed in each wound. No difference of the thickness was observed between the two treatment groups. (E) No difference in VEGFa cytokine expression was found between the two

treatment groups (F) Macrophages detected by antibody B4AD5 with flow cytometry shows no significant difference measured between DEVICE and SOC.

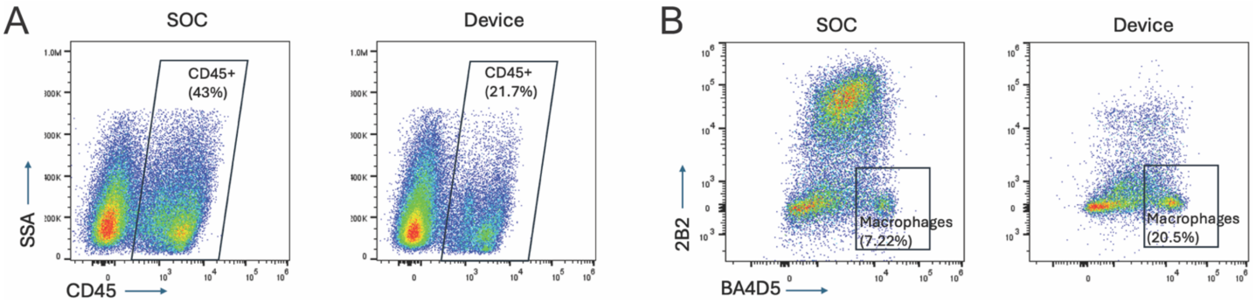

Figure S4. (A) Representative flow cytometry quantification of immune cell presence. (B) Representative flow cytometry quantification of macrophages.

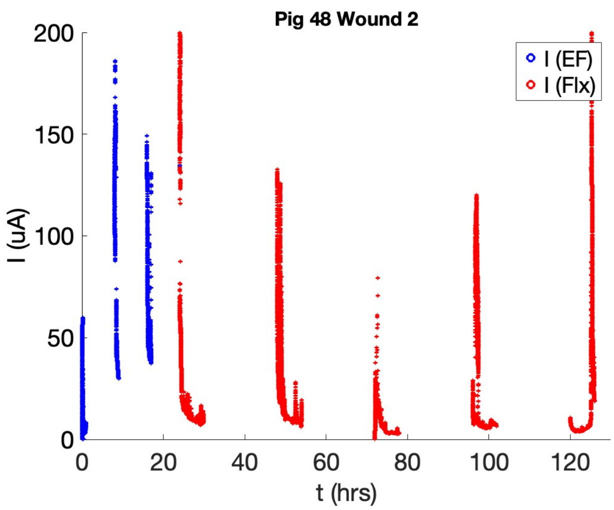

Figure S5. In vivo current delivery data from a single characteristic device-treated wound collected on the SD card of the control board. Current data was recorded as average peak of pulse.

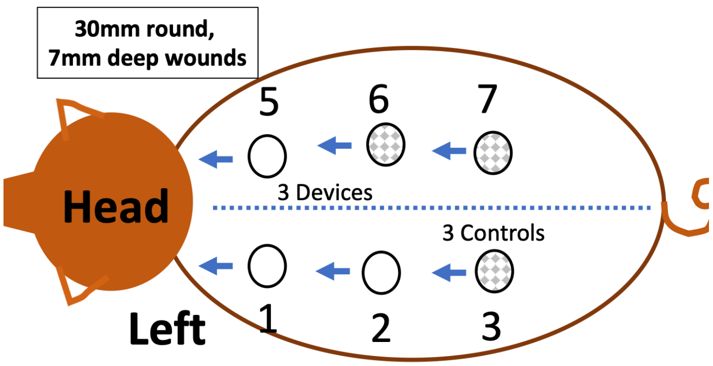

Figure S6. Porcine wound and treatment locations.

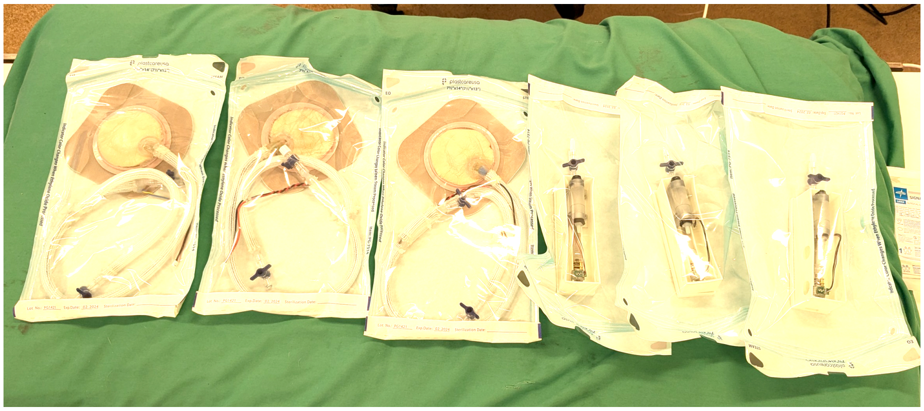

Figure S7. Bioelectronic dressings (left), and pump modules (right), in packaging prior to in vivo application.

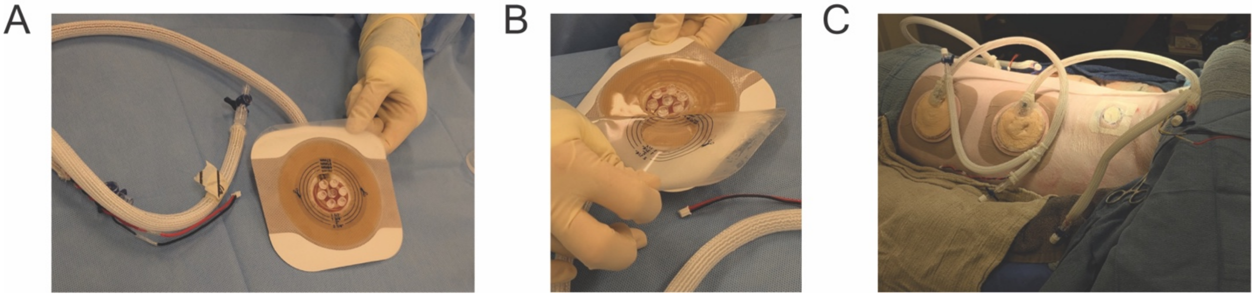

Figure S8. (A) Bioelectronic dressing before wound application. (B) Removal of release liner. (C) Application of bioelectronic dressings to wound

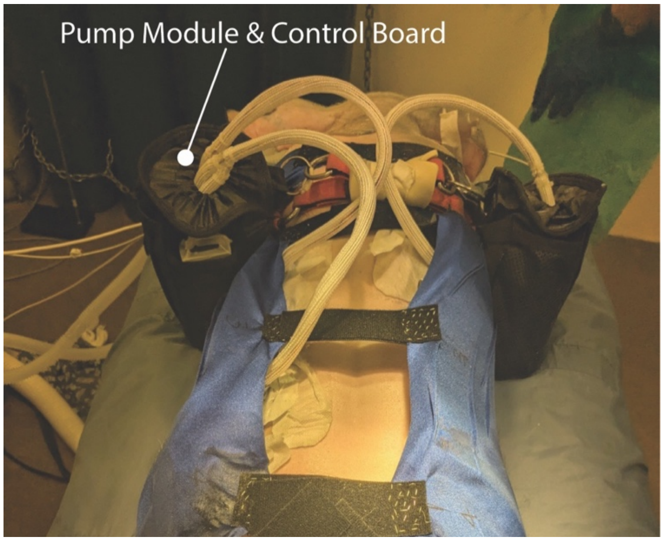

Figure S9. Photo depicts pump module and control board location inside of harness bags carried by porcine model for duration of treatment with active dressing.

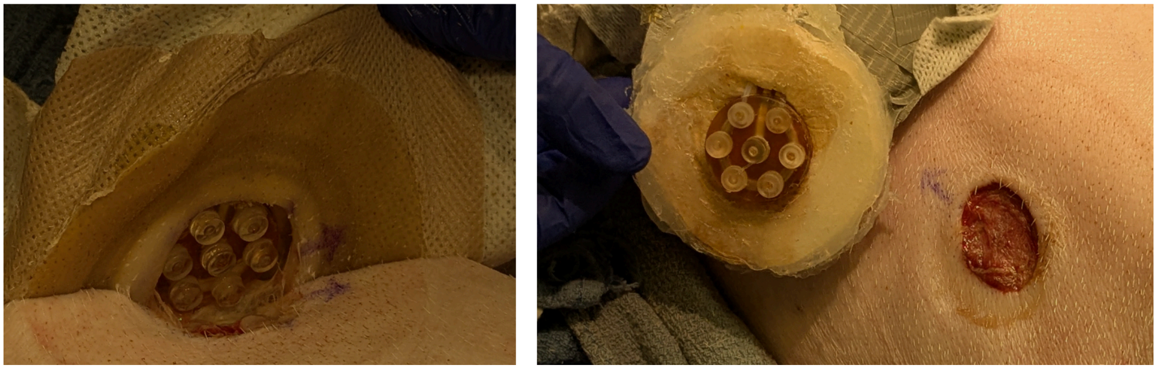

Figure S10. Post-operative day 6 bioelectronic dressing removal.

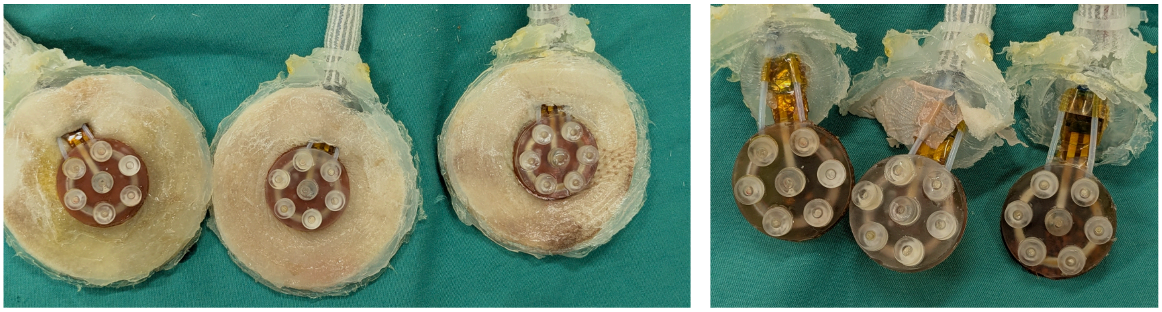

Figure S11. Removed post-operative day 6 bioelectronic dressings.

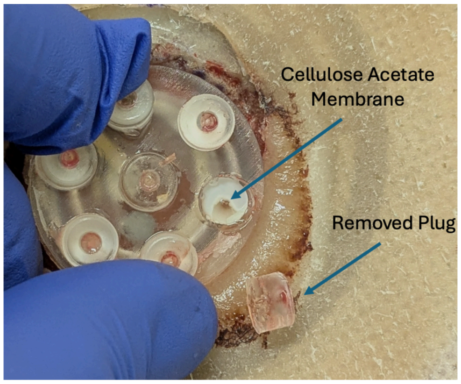

Figure S12. Absorption of wound exudate in cellulose acetate membrane of deconstructed device after 3-day in vivo deployment.

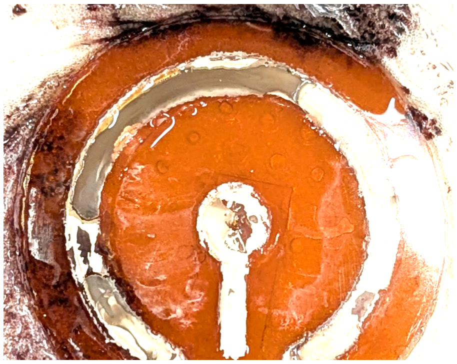

Figure S13. Chlorination of outer working electrode observed in deconstructed device 3-day in vivo deployment.

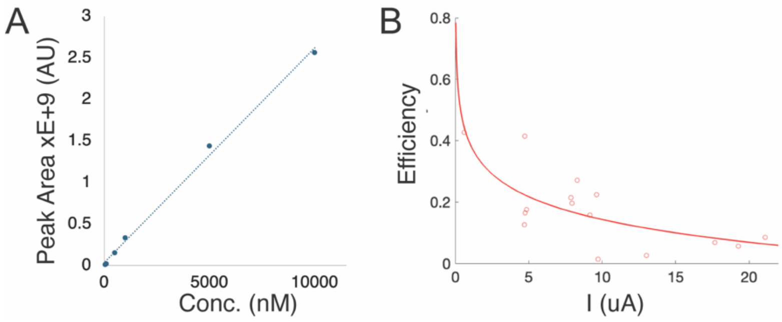

Figure S14. (A) HPLC calibration function ($y = 258540x + 4E07, R^2 = 0.996$) derived from fluoxetine standard solution aliquots (B) Actuator current efficiency (the proportion of total charge, calculated by integrating current, detected as Flx by HPLC analysis) as a function of measured current (n=15). The current efficiency function from the fitted curve ($y = -0.1069\, ln(x) + 0.3895, R^2 = 0.55$), is used to calculate rate of active drug delivery.

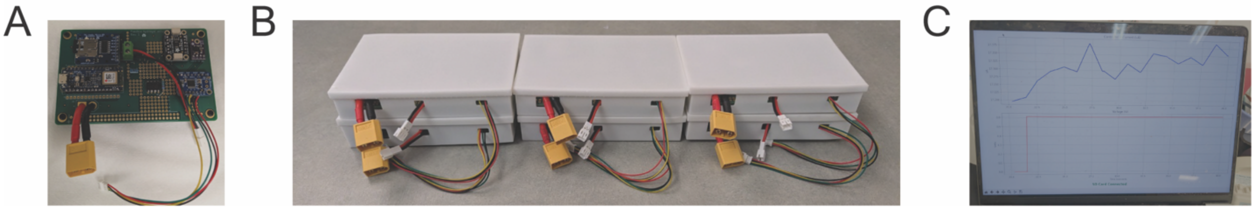

Figure S15. (A) Control electronics PCB. (B) Control boards in 3D printed enclosures. (C) Python program displaying real-time current, voltage, and SD card status data sent from the control board via BLE connection.

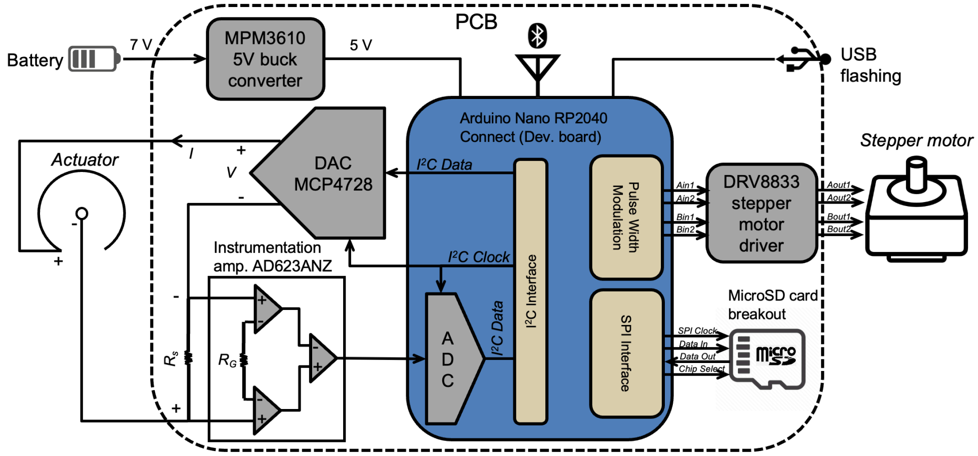

Figure S16. Detailed control board diagram.

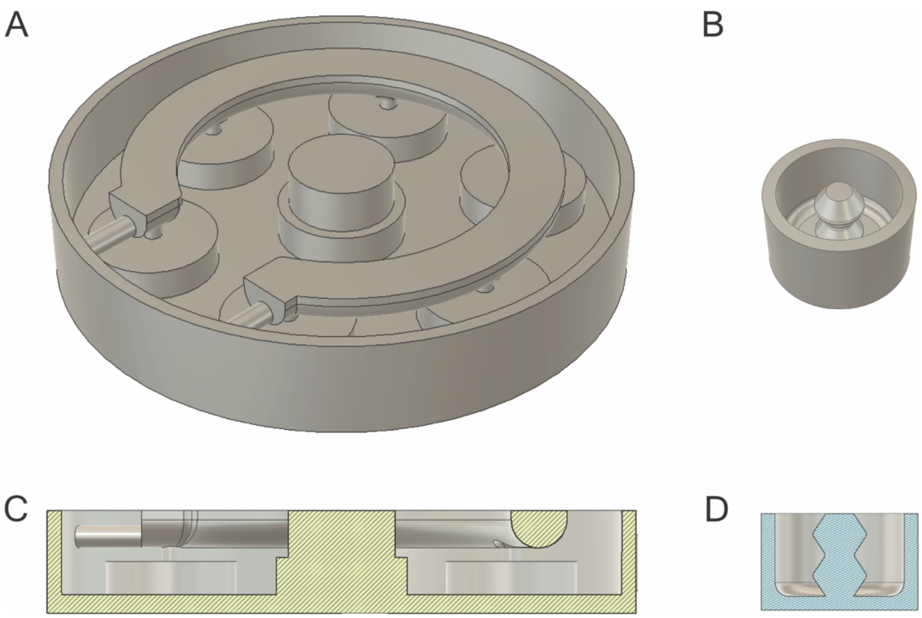

Figure S17. (A) CAD model of PDMS mold for dressing actuator main body. (B) CAD model of mold for PDMS protrusions. (C) Cross section of main body mold. (D) Cross section of PDMS protrusion.

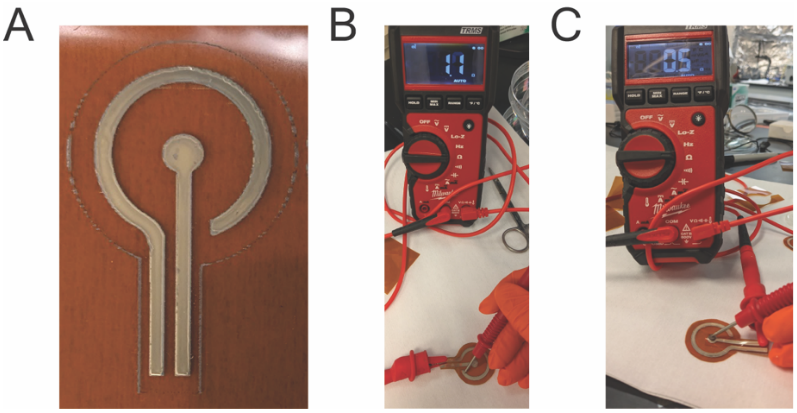

Figure S18. (A) WE and CE trace printed on polyimide. (B) Resistance testing of WE trace. (C) Resistance tracing of CE trace with inserted AgCl pellet electrode.

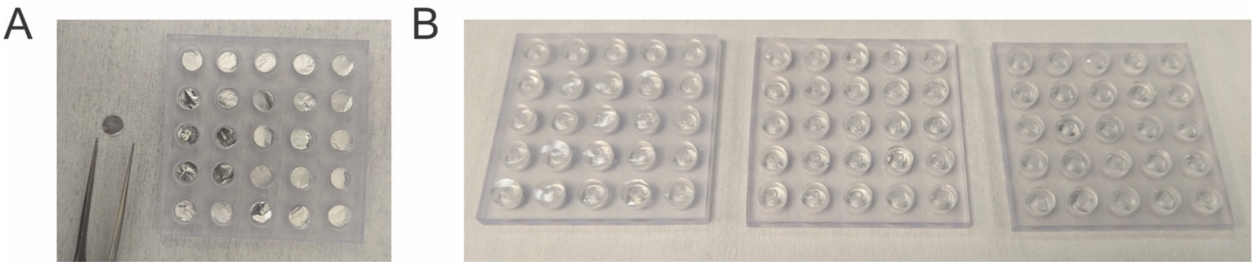

Figure S19. (A) Plug curing fixture with 6mm punched aluminum foil disks. (B) Plug curing fixture with PDMS protrusions inserted.

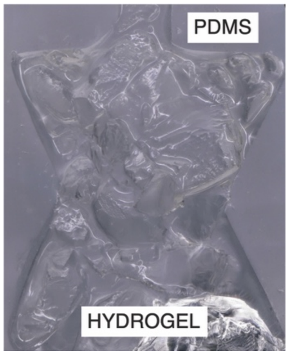

Figure S20. Bisected PDMS protrusion with internally cured double fluted hydrogel plug test pattern.

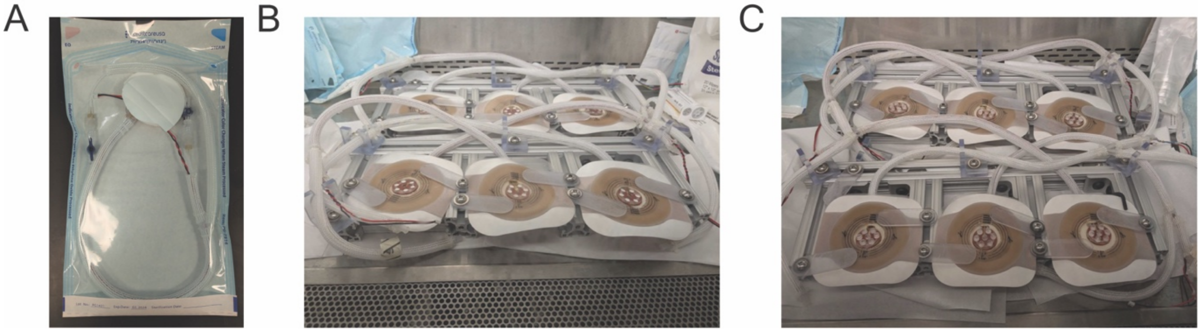

Figure S21. (A) Autoclaved upper assembly in autoclave bag (B) Ostomy barrier attachment and cellulose acetate membrane addition to dressing upper assembly with fabrication jig inside biosafety cabinet. (C) PDMS protrusions with hydrogel plug insertion to dressing upper assembly.

Device class	Therapeutic modalities	Modality combination	Mechanism	Clinical / translational status	Comparison to this work
Standard of care dressings[1]	Passive protection, moisture control, absorption	Single, static function	No active mechanism	Clinical, widely used	No active therapy or programmability
Negative pressure wound therapy [2]	Mechanical suction	Single modality	External vacuum pump	Clinical, widely used	Mechanical conditioning only
Clinical electrical stimulation [3]	Electric field / microcurrent	Single modality	External electrodes or preset patch circuitry	Clinical	EF only, no drug delivery
Passive Electroceutical dressings [4]	Passive microcurrent	Single modality	Self-generated electrochemical potential	Clinical	Fixed, non-programmable output
Smart bandages with sensing & stimulation [5]	Monitoring, stimulation, & delivery	Parallel functions	Integrated sensors & electronics	Translational	Focus on sensing
Drug-eluting wound dressings [6]	Pharmacologic delivery	Single modality	Passive diffusion or triggered release	Clinical / translational	No EF, rate of delivery typically governed by diffusion
Bioelectronic drug-delivery dressings [7]	Drug delivery	Single modality	Current-driven ion transport	Translational	Drug based therapy only, often single drug
This work	Electric field & iontophoretic drug delivery	Sequential	Pump-driven microfluidic solution exchange feeding a single ion-selective actuator	Preclinical	NA

Table S1. Comparison between the work presented in this paper to relevant established and emerging wound healing devices and technologies.

SUPPLEMENTARY TEXT S1

Bioelectronic Dressing Fabrication

Molds for the actuator main body and hydrogel plug protrusions were made by first printing molds using Form 3B printers using Formlabs Model V3 Resin. PDMS (Sylgard 184) with a base to cross-linker of 10:1. After mixing, the PDMS is degassed to remove trapped air. The PDMS is then poured into the 3D printed molds. The molds are carefully leveled sing a glass slide and cured at 60°C for 48 hours. After curing, the resin printed connection between the fluidic channel, plug insert locations, and tubing connection as well as the side wall of the mold are cut with pliers to allow for demolding without ripping internally molded features. The PDMS components are sonicated in isopropanol (IPA) for 15 minutes, then sonicated with DI water for 15 minutes and allowed to dry.

Electrodes were inkjet printed (Epson XP-15000) using NovaCentrix, JS-A010ET Silver Nanoparticle Ink, 30%w/w Ag ink on a polyimide substrate and cured at 250C for 2 minutes. Next an Ag/AgCl pellet (World Precision Instruments EP4) is inserted into the CE trace of the electrodes. PDMS main actuator bodies were then cut out of their molds and bonded to the electrodes using an ISO-10993 compliant epoxy (Loctite M-31CL). A 5 kg weight was then placed on top of the bonded electrode and allowed to cure for 24 hours. The tail of the electrode trace was than attached to a FFC connector (DigiKey 609-2158-ND) for integration with harness wiring. PTFE tubing is then inserted into the main body channel connection (Darwin Microfluidics BL-PTFE-1608-50) and wiring attached to the FFC connector. Silicone wound dressing foam (Dimora) was in a 73mm diameter circle with a slit cut through it for actuator tubing and wiring to pass. Epoxy is applied to the back of the actuator and is adhered to the adhesive side of the wound dressing foam. The wires and tubing of the actuator run through a 3D printed (Formlabs Biomed Clear Resin) sleeve port connector. The face of the tubing port connector is attached to the wound dressing foam using silicone adhesive and a custom fabrication jig. A heat-resistant braided sleeve (TECHFLEX) was then run over actuator tubing and wiring. The sleeve is attached to the other end of the sleeve port connector with more silicone adhesive and heat-resistant zip ties. A 3D printed sleeve splitter is then applied with more sleeve material to separate the inlet tubing and electrode connection from the outlet tubing for attaching to the pump module. Microfluidic tubing connectors (Darwin Microfluidics) are attached to the bioelectronic dressing tubing to enable connection with the pump module. This upper assembly of the dressing is then autoclaved on the solid setting for 1hr inside autoclave bags.

The anionic hydrogel used as our ion exchange membrane consists of 2-acrylamido-2-methylpropane sulfonic acid (AMPSA) and poly(ethylene glycol) diacrylate (PEGDA) and a UV photo initiator (2-hydroxy-4'-(2-hydroxyethoxy)-2-methylpropiophenone).Protocols and characterization of this hydrogel have been previously reported.[8] Demolded PDMS protrusions are then demolded and placed in a resin printed curing fixture (Formlabs BioMed Clear Resin). 20uL of hydrogel is then injected into the protrusions and given 4J of UV energy inside a UV crosslinker (Boekel). This process is repeated, progressively curing hydrogel in the double fluted plug shape molded in the PDMS protrusion. Because the hydrogel would not remain stable in an autoclave this process (in addition to integration with the upper assembly) is conducted inside a thoroughly sterilized biosafety cabinet.

The autoclaved upper assemblies next brought into the biosafety cabinet. To achieve enhanced adhesion required for multi day use in a porcine model the upper assembly is integrated into an ostomy barrier selected for its adhesive properties. A 38 mm diameter circle is cut out of the center of the barrier ensuring that only the actuator or the wound dressing foam has any possibility of directly contacting the wound area. The actuator is passed through the hole of the ostomy barrier, and the foam of the upper assembly is bonded to the back of the ostomy barrier with more medical grade epoxy and held in place using a custom fabrication jig while drying. Next, 0.2um pore, 6 mm diameter, cellulose acetate membrane disks are inserted into the bottom of the dressing actuator, below which each PDMS protrusion will sit. The cellulose acetate membrane additionally serves to prevent entry of wound exudate, that may coagulate and clog the fluidic channel, from entering the microfluidic system.

The PDMS protrusions with cured hydrogel plugs are then glued into the actuator body by carefully applying epoxy to the walls of the protrusion insertion cavities. UV light from the biosafety cabinet is applied for the first 2 hours of curing for sterility. A pipette is used to apply droplets keep hydrogel plugs hydrated while the epoxy dries. After the epoxy has fully cured, the fluidic tubing of the dressing is filled and capped. The fixed CE electrolyte reservoirs were filled with solution (NeilMed Wound Wash) by injection with a 22g needle. The actuators are then fitted with 3D printed autoclaved transport holder filled with solution, and the entire dressing is sealed in sterile packaging for transport.

Pump Module Fabrication

The pump box, box top, plunger arm, and dual chamber reservoir is 3D printed using Formlabs biomed resin. Threading for fluidic connections on the reservoir is hand tapped. The pump module is the assembled from the resin printed parts, luer connectors, microfluidic connectors (Darwin Microfluidics), a M5 breather vent (McMaster-Carr), the piston from an insulin pump reservoir (Medtronic MiniMed 3.0mL), and stainless steel M3 screws and square nuts (McMaster-Carr). The pump module is then sealed in autoclave bags and autoclaved for 1 hour. The pump modules are then brought into the biosafety cabinet and the supply reservoir chamber is filled with 10mM FlxHCl through a 0.22um filter. The inlet and outlet connections of the supply reservoir are then capped, and the pump module is sealed in sterile packaging for transport.

Control Board Fabrication

The control board is made from a custom PCB (Sierra Circuits), an Arduino Nano 33 BLE (DigiKey 1050-ABX00072-ND), an instrumentation amplifier (DigiKey AD623ANZ-ND), a buck converter (MPM3610), a DAC (MCP4728), a MicroSD card breakout board (Adafruit Industries), and a stepper motor driver (DRV8833). Each control board was tested and individually calibrated

using resistors after soldering. Control boards are then inserted into a 3D printed enclosure (Formlabs Biomed White).

Control Board Software

The control board is programmed to deliver specific currents during pre-programmed time intervals respective to each therapy type. Target currents are achieved by varying electrode voltage using PID control. Due to sudden rapid changes in contact resistance due to application of the device on a mobile and active animal, as well as the highly variable nature of both wounds and electrochemistry, the control function implemented is purposely overdamped. This reduces the likelihood that unpredictable short-term fluctuations in control input will send the system into a sustained oscillatory pattern. To reduce interface capacitance by disrupting the buildup of charge at the electrode-electrolyte interface, and sustain electrode performance over long time periods, the controller pulses its voltage output at a rate of 0.86Hz with a 77% duty cycle. Current values are read and averaged during the peak of the pulse and the average current of peak, control voltage, and system time are recorded to the microSD card once per cycle. In addition to recording these values locally, the control board also transmits these values, as well as microSD card status over BLE. A corresponding python program was written to view the current, voltage, and microSD status data from the board in real-time. This allowed device performance to be assessed immediately after attachment to the wound, enabling intervention in the event of malfunction or poor wound contact.

Tissue Sample Histology

Tissue samples were collected immediately after pigs were euthanized on day 22. Statistical significance was determined using the two-tailed Mann Whitney U test.

qPCR

Wound tissue was preserved in Invitrogen RNALater Stabilization Solution (Fisher Scientific) at 4 degree for 4 days and frozen at -80 degree according to the manufacturer's instructions. The frozen tissue was trimmed with a 5mm biopsy punch from the wound edge (~50 mg tissue), minced to 1mm pieces, and homogenized with Tissue Tearor (BioSpec cat# 98537004) in 300 µl Buffer RLT containing β-mercaptoethanol (Qiagen RNeasy Fibrous Tissue Mini Kit) on ice for 2 minutes. The mRNA was extracted with Qiagen RNeasy Fibrous Tissue Mini Kit, and cleaned with DNase I according to the manufacturer's instructions. One µg of RNA was reverse transcribed to cDNA using the Quantitect Reverse Transcription Kit (Qiagen).

The following Qiagen RT2 qPCR Primer for pigs were used: Pig IL1B2 (PPS00015A-200), Pig IL6 (PPS00991A-200), Pig IL10 (PPS00445B-200), Pig IGF1 (PPS00801A-200), Pig TGFB1 (PPS00418A-200), Pig TNF (PPS00426A-200), Pig VEGFA (PPS00495A-200), Pig RPL19 (PPS00333A-200), and Pig GAPDH (PPS00192A-200). For qPCR analysis, the Quantitect Sybr Green master mix was used (Qiagen) on a 384-well platform (Bio-Rad CFX384 Real-Time System, C1000 Touch Thermal Cycler). Each sample was run in triplicate and mRNA levels of target genes were normalized to the average levels of housekeeping genes GAPDH and RPL19. Relative expression of the target gene was calculated via ΔΔCt against the average Ct of the housekeeping genes and the average Ct of day 0 skin from each animal.

Cytokine and growth factor values were compared between standard of care (SOC, n= 6/group from 2 pigs) and device-treated wounds (n= 5/group from 2 pigs, one wound was excluded due to device detachment). Data were analyzed using both multivariate and univariate approaches. Multivariate analysis of variance (MANOVA) and permutation-based multivariate analysis of variance (PERMANOVA, 999 permutations, Euclidean distance) were used to assess global differences across the cytokine panel. For individual cytokines, two tailed Mann-Whitney U tests were performed, and exact two-sided permutation tests (all possible label permutations, n=924) were used to obtain small-sample p-values. To account for multiple comparisons, p-values were adjusted using the Benjamini–Hochberg false discovery rate (FDR) procedure. Effect sizes (Cohen's d) were calculated from group means and pooled standard deviations. Given the limited sample size (n= 5 or 6/group), the study was underpowered to detect modest effects, and results are interpreted as trending, exploratory and hypothesis-generating.

Isolation of Single Cells

Following tissue harvest, the fat layer and excess healthy tissue was trimmed and discarded, leaving a 3 mm rim of healthy skin at the wound edge. The skin was weighed and up to 500 mg of tissue was transferred to a 60 mm tissue culture dish with 5 mL of digestion media (cRPMI (RMPI supplemented with 10% FBS , 1mM sodium pyruvate, 0.1mM non-essential amino acids, and 100U/ml penicillin/streptomycin) containing 1.66 mg/mL Dispase II, 9.6 mg/mL Collagenase Type 4, 2 mg/ml DNase I), sliced into small strips, and diced into pieces of less than 2mm using a steel razor blade (AccuTec Blades #940120).

The tissue was incubated at 37°C with 5% CO_2 for 2 hours. Plates were agitated every 15 minutes. The suspension was triturated with a 2ml pipette and was pushed through a 100μm cell strainer with the back of a 3mL syringe plunger. The cell suspension was passed through a second 100μm strainer and centrifuged at 524RCF at 4°C for 5 minutes. The cells were then resuspended in 40% Percoll in cRPMI and overlayed onto an equal volume of 70% Percoll in PBS and centrifuged in a swinging bucket centrifuge at 1825RCF at 4°C for 20 minutes without brake. Cells were collected from the interface, washed with 10x volume of 1X PBS centrifuged at 524RCF at 4°C for 5 minutes, and resuspended in cRPMI.

For the isolation of single cells from the Optifoam, the Optifoam was washed with cRPMI and cells were pushed from it using the back of a plunger of a 3 ml syringe. Cells passed through a 100µm cell strainer. Single cells were isolated from the interface of a 40/70% Percoll as described above.

Flow Cytometry

Cells were plated in a 96 well staining plate and centrifuged at 524RCF at 4°C for 5 minutes. Viability dye (Zombie Red Biolegend) was added, and cells were incubated on ice for 25 minutes, then washed with 200uL of FACS buffer (DPBS containing 4mg/mL BSA and 2mM EDTA) and centrifuged at 524RCF at 4°C for 5 minutes. Cells were incubated with extracellular antibodies overnight at 4°C in the dark. The next day, cells were washed with 200uL of FACS buffer and centrifuged at 524RCF at 4°C for 5 minutes. After fixation and permeabilization (Cytofix/Cytoperm Buffer, BD Biosciences) cells were incubated with intracellular antibodies in permeabilization buffer and incubated on ice and in the dark for 30 min. After washing, cells were resuspended in 200 uL FACS buffer and analyzed on an Attune NxT Flow Cytometer. Data were analyzed using FlowJo Software. Antibodies used were: CD45 for all immune cells (Bio-Rad MCA1222PB), 2B2 for granulocytes (expected to be mostly neutrophils; Bio-Rad MCA2600F), and BA4D5 for macrophages (Bio-Rad MCA2317A647). Statistical significance was determined using the one-tail Mann Whitney U test.

EF Strength calculation

Estimated induced EF strength was obtained by simulation using COMSOL. A CAD model of actuator geometry was uploaded on top of a 5 mm thick layer as tissue with the conductivity of 0.17 S/m, which is an average of muscle layer at different directions [9]. The simulation calculated both voltage distribution and wound resistance. In vivo average EF strength is calculated using wound resistance, current data captured by the control board, the center-to-center distance of the WE hydrogel plugs to the CE plug, and the number of hydrogel plugs connected in parallel.

$$E_{avg} = \frac{I \cdot R}{d * n}$$

E_{avg} is the average exogenously induced electric field strength from WE to CE hydrogel plugs (mV/mm),

I is the recorded device current (uA)

R is the wound resitance resistance: 31.1 kΩ.

d is the distance: 10.5 mm

n is the number of hydrogel plugs connected in parallel: 6

SUPPLEMENTARY VIDEO S1

Supplementary video S1 shows pumping of two different colored solutions into the flexible bioelectronic actuator, representing fluids associated with two distinct treatment modalities.

REFRENCES

1 Dhivya, S., Padma, V. V. & Santhini, E. Wound dressings - a review. *Biomedicine (Taipei)* **5**, 22 (2015). https://doi.org/10.7603/s40681-015-0022-9

2 Panayi, A. C., Leavitt, T. & Orgill, D. P. Evidence based review of negative pressure wound therapy. *World Journal of Dermatology* **6** (2017). https://doi.org/10.5314/wjd.v6.i1.1

3 Rajendran, S. B., Challen, K., Wright, K. L. & Hardy, J. G. Electrical Stimulation to Enhance Wound Healing. *J Funct Biomater* **12** (2021). https://doi.org/10.3390/jfb12020040

4 Chan, R. K. *et al.* A Prospective, Randomized, Controlled Study to Evaluate the Effectiveness of a Fabric-Based Wireless Electroceutical Dressing Compared to Standard-of-Care Treatment Against Acute Trauma and Burn Wound Biofilm Infection. *Adv Wound Care (New Rochelle)* **13**, 1-13 (2024). https://doi.org/10.1089/wound.2023.0007

5 Derakhshandeh, H., Kashaf, S. S., Aghabaglou, F., Ghanavati, I. O. & Tamayol, A. Smart Bandages: The Future of Wound Care. *Trends Biotechnol* **36**, 1259-1274 (2018). https://doi.org/10.1016/j.tibtech.2018.07.007

6 Vilangattu Parambu Kunjikuttan, R., Jayasree, A., Biswas, R. & Jayakumar, R. Recent developments in drug-eluting dressings for the treatment of chronic wounds. *Expert Opin Drug Deliv* **13**, 1645-1647 (2016). https://doi.org/10.1080/17425247.2016.1238456

7 Hossain, M. I. *et al.* Smart bandage: A device for wound monitoring and targeted treatment. *Results in Chemistry* **7** (2024). https://doi.org/10.1016/j.rechem.2023.101292

8 Jia, M., Luo, L. & Rolandi, M. Correlating Ionic Conductivity and Microstructure in Polyelectrolyte Hydrogels for Bioelectronic Devices. *Macromol Rapid Commun* **43**, e2100687 (2022). https://doi.org/10.1002/marc.202100687

9 Gabriel, C., Peyman, A. & Grant, E. H. Electrical conductivity of tissue at frequencies below 1 MHz. *Phys Med Biol* **54**, 4863-4878 (2009). https://doi.org/10.1088/0031-9155/54/16/002
